# Free flap versus pedicled flap reconstruction in head and neck surgery: A systematic review and meta-analysis

**DOI:** 10.1016/j.jpra.2026.07.021

**Published:** 2026-07-16

**Authors:** Lara Crouch, Katharine Fleming

**Affiliations:** a Gold Coast University Hospital, Australia; b The Royal Brisbane and Women’s Hospital, Australia

**Keywords:** Reconstructive surgical procedure, Free flaps, Pedicled flap, Surgical flaps

## Abstract

**Background:**

Free and pedicled flaps remain the principal reconstructive options following head and neck oncologic surgery. Although free flaps are most frequently used, pedicled flaps have re-emerged with modern techniques offering comparable outcomes with reduced complexity. The aim was to systematically compare surgical, functional and economic outcomes between free and pedicled flaps in head and neck reconstruction.

**Methods:**

A systematic search of *PubMed* and *Embase* was conducted from database inception to September 2025, following PRISMA guidelines. Comparative studies evaluating free versus pedicled flap reconstruction in adults were included. Two reviewers independently screened and extracted data. Pooled meta-analyses were performed for operative time, hospital stay and complications.

**Results:**

Twenty-five studies met inclusion criteria. Free flap reconstruction required a longer operative time (mean difference = +172.5 min; 95% CI 132.3–212.6, PI 2.5 to +342.5 min) and higher financial cost (1.3–3 × greater) compared with pedicled flaps. Hospital stay was similar between groups (mean difference = +0.6 days; 95% CI -2.7 to +3.9). Pedicled flaps exhibited slightly fewer overall complications, including wound dehiscence, infection and revision surgery. Flap failure was significantly more frequent with free flaps.

**Conclusions:**

Free flaps remain the standard for extensive or functionally demanding reconstructions, but pedicled flaps offer comparable outcomes with shorter surgery, lower costs and equivalent complication rates in selected cases. Flap choice should be individualised based on defect complexity, patient comorbidity and institutional expertise.

## Introduction

Reconstructive surgery is an essential component of managing oncologic and traumatic defects in the head and neck. Historically, pedicled regional flaps, most notably the pectoralis major myocutaneous flap (PMMF), served as the workhorse for reconstruction in this region.^1^ With the advent of microsurgery in the late 20th century, free tissue transfer largely supplanted pedicled flaps as the preferred technique for complex defects.^2^ Free flaps, such as the radial forearm free flap (RFFF), fibula and anterolateral thigh (ALT) flaps, enable the transplantation of well-vascularised tissue to the defect site via microvascular anastomosis. In contrast, pedicled flaps (e.g. PMMF, supraclavicular artery island flap (SCAIF), submental island flap (SMIF)) are raised with an intact vascular pedicle and rotated or tunnelled into position.

Each reconstructive strategy offers distinct advantages. Free flaps provide pliable, well-vascularised tissue that can be tailored to the three-dimensional needs of the defect, often resulting in superior aesthetic and functional outcomes.^3^ However, these procedures are resource-intensive, requiring microsurgical expertise, longer operative times and dedicated postoperative monitoring.^1^ They also carry a risk of complete flap loss due to vascular thrombosis. Pedicled flaps avoid microvascular anastomosis, allowing shorter operative times and reduced perioperative demands. They are particularly advantageous in patients with significant comorbidities or in resource-limited settings.^1^ The trade-off, however, is that traditional pedicled flaps may be bulkier, have limited reach and have been associated with higher rates of partial necrosis or wound complications.

Despite the widespread adoption of free flaps as the gold standard for major head and neck defects, their superiority across all contexts remains debated.^2^ Advances in regional flap design, particularly with the SCAIF and SMIF, have narrowed the gap between pedicled and free flaps, demonstrating comparable reconstructive and functional outcomes for small to medium defects while offering shorter operative times and lower costs.^1^^,^^6^ Additionally, pedicled flaps may be preferable in elderly or medically frail patients who are less suited to prolonged anaesthesia, even if free flaps yield slightly better reconstructive precision. From a health economics perspective, the substantially higher costs associated with free flap surgery may not always be justified when pedicled alternatives can achieve satisfactory results with fewer resources.^1^

Multiple comparative studies have examined free versus pedicled flap outcomes, but most have been limited by small sample sizes, variable patient selection and heterogeneity in flap types and anatomical sites. Earlier retrospective analyses reported that pedicled flaps were not significantly inferior to free flaps in overall success and function,^4^ whereas recent large database studies suggest free flaps may reduce specific complications after adjusting for patient factors.^2^ However, consensus remains lacking regarding which approach offers superior clinical or economic outcomes.

To address this gap, we conducted a comprehensive systematic review and meta-analysis comparing free and pedicled flap reconstruction in head and neck surgery. Our objectives were to synthesise current comparative evidence, evaluate differences in operative time, hospital stay, cost and complication rates and assess methodological quality using validated bias tools. By pooling data from studies published up to September 2025, this review provides the most up-to-date and quantitative comparison of these two reconstructive approaches, aiming to guide evidence-based flap selection and optimise outcomes for head and neck surgical patients.

Largely composed of retrospective cohort studies the available literature is inherently vulnerable to selection bias, confounding and inconsistent reporting. This is further compounded by the heterogeneity of patient pathology, defect characteristics and reconstructive indications. Whilst a prospective randomised trial would minimise these biases, the design would raise significant ethical concerns in the field of surgical reconstruction. Thus, adhering to the parameters of available evidence, this review aims to provide up to date guidance while acknowledging the methodological constraints and inherent bias within current literature.

## Methods

### Protocol and registration

This systematic review and meta-analysis adhered to the Preferred Reporting Items for Systematic Reviews and Meta-Analyses (PRISMA) guidelines.^5^ A formal PROSPERO protocol was published (#CRD420251149544).

### Eligibility criteria

Comparative studies directly evaluating free flap versus pedicled flap reconstruction in head and neck surgery were included. Inclusion criteria were as follows:

• **Population:** Human patients undergoing reconstructive surgery for head and neck defects, most commonly following oncologic resection but also including trauma or other causes where reported.
• **Intervention and Comparator:** Studies comparing microsurgical free tissue transfer with regional or pedicled flaps. Studies without a direct comparison between the two were excluded.
• **Outcomes:** Studies reporting at least one relevant outcome (operative time, hospital length of stay (LOS), cost or postoperative complications) were included.
• **Study design:** Eligible designs included randomised controlled trials and observational comparative studies. Case series without comparators and single-arm studies were excluded.
• **Language and Timeframe:** Only studies published in English were included. Searches were conducted from database inception to September 2025.

### Information sources and search strategy

A systematic literature search was conducted in PubMed (MEDLINE) and Embase from inception to 15 September 2025. Search terms combined concepts for head and neck reconstruction, free flaps and pedicled or regional flaps. A representative PubMed search strategy was:

(“free flap” OR “free tissue transfer” OR “microsurgery”) AND (“pedicled” OR “regional flap” OR “local flap” OR “pectoralis” OR “supraclavicular” OR “submental”) AND (“head neck” OR “oral” OR “oropharynx” OR “mandibular” OR “larynx”)

**Fig. 1:**
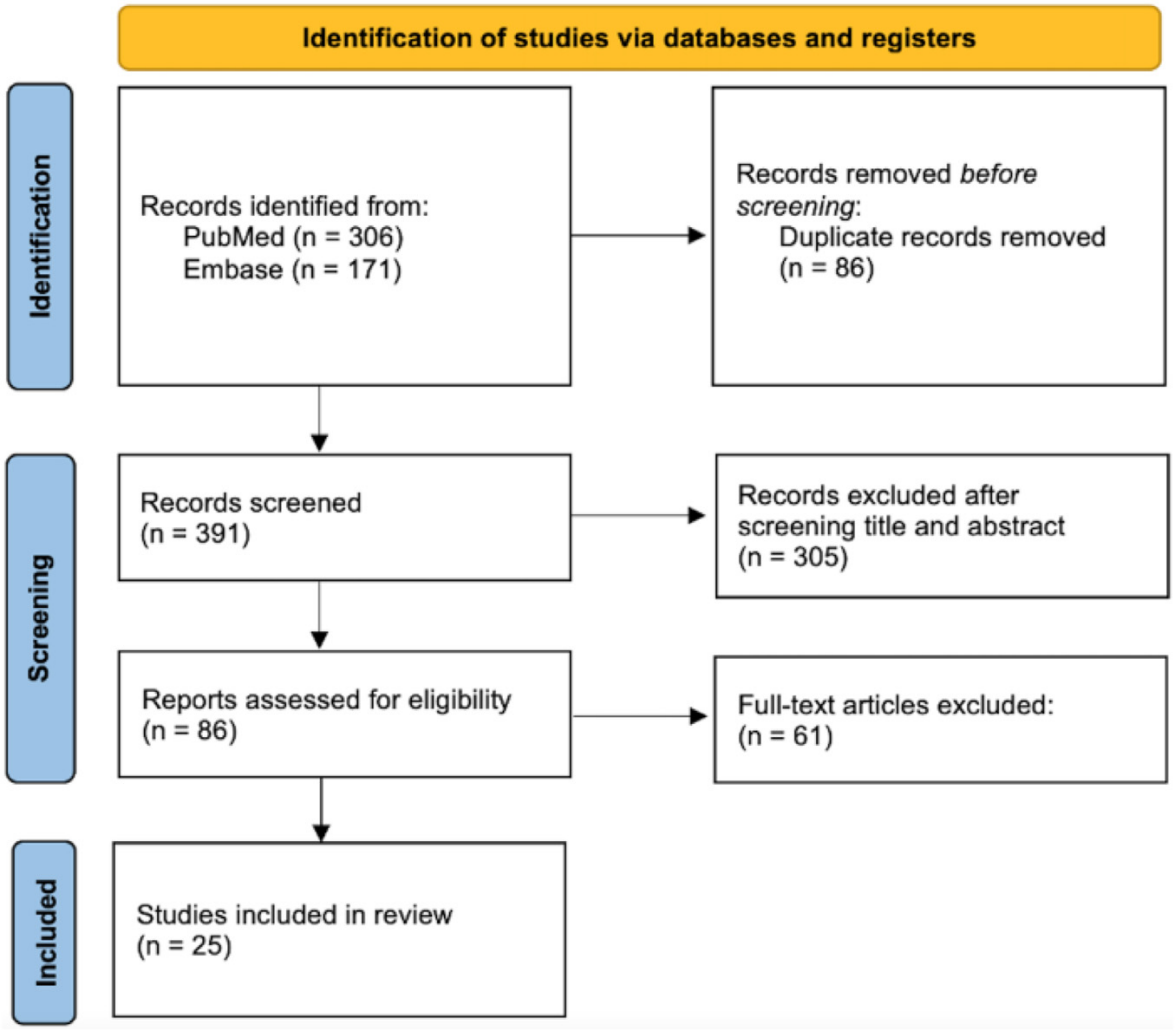
PRISMA flow diagram.

Reference lists of included studies and previous reviews were manually screened to identify additional eligible articles. After removal of duplicates, two reviewers independently screened all titles and abstracts, followed by full-text assessment for inclusion. Discrepancies were resolved through discussion until consensus was reached. A PRISMA flow diagram (Fig. 1) summarises the selection process.

### Data extraction

Two reviewers independently extracted data using a standardised form. Extracted information included study characteristics (author, year, design and sample size), patient demographics, type of flap used and all relevant outcomes (operative duration, LOS, cost and complications) (Table 1). For complication data, the incidence of specific events (e.g. fistula formation, infection) was recorded separately for free and pedicled flap cohorts. Disagreements were resolved through review and discussion. Studies with unclear or missing quantitative data were excluded from pooled analysis.

**Table 1:** Characteristics of included articles.

Source	Type of Review	Total pts	N Free Flap	N Pedicle Flap	Mean age	No females	No Males
Al-Aroomi et al. (2025)^7^	Retrospective	37	21	16	49.62	14	23
Benazzo et al. (2006)^8^	Retrospective	67	53	14	58.9	3	64
Brown et al. (2009)^9^	Retrospective	245	130	115	59.98		
Chen et al. (2005)^10^	Retrospective	77	28	49	49.95	1	76
Clark et al. (2009)^11^	Retrospective	132	68	64	62	35	118
Escalante et al. (2021)^12^	Retrospective	122	68	54	70.5	31	91
Forner et al. (2016)^13^	Retrospective	21	12	9	64	8	13
Gonzalez-Garcia et al. (2021)^14^	Retrospective	31	15	16	63.74	10	21
Haidar et al. (2018)^15^	Retrospective	347	204	143	62.5	71	276
Hanasono et al. (2012)^16^	Retrospective	117	90	27	64.1	22	95
Howard et al. (2016)^17^	Retrospective	31	9	22	73.5	3	28
Jang et al. (2013)^18^	Retrospective	25	11	14	64	3	22
Katna et al. (2021)^19^	Retrospective	628	481	147	49	89	539
Katsnelson et al. (2021)^2^	Retrospective	1505	1297	208	61.2		
Mahieu et al. (2016)^20^	Retrospective	93	29	64	62.54	31	62
McCrory, Magnuson (2009)^21^	Retrospective	59	34	25	61.2		
Padha et al. (2022)^22^	Retrospective	115	25	90	54.1	5	110
Patel (2020)^23^	Retrospective	146	89	57	56.19	48	98
Paydarfar et al. (2011)^24^	Retrospective	60	33	27	56.23	18	42
Pradhan et al. (2021)^25^	Retrospective	33	8	25	41.47	12	32
Ratnagiri et al. (2018)^26^	Retrospective	265	86	179	56.5	106	220
Sittitrai et al. (2023)^27^	Retrospective	171	73	98	62.1	54	117
Sittitrai et al. (2024)^28^	Retrospective	146	94	52	52.8	49	84
Spoerl et al. (2019)^29^	Retrospective	494	126	368	62	177	315
Sun et al. (2025)^30^	Retrospective	147	43	104	54.71	29	118

### Data synthesis and statistical analysis

A qualitative synthesis was first performed to summarise findings across studies. For each outcome, the direction of effect and statistical significance were noted. When at least three studies reported comparable quantitative data for an outcome, a meta-analysis was undertaken. For binary outcomes (e.g. complications), pooled risk ratios (RR) with 95% confidence intervals (CIs) were calculated. For continuous outcomes (e.g. operative time, LOS, cost), pooled mean differences (MD) were computed using reported means and standard deviations. When studies reported medians and ranges, these were converted to approximate means and SDs using established methods.

Given expected clinical and methodological heterogeneity, all analyses used a random-effects model (DerSimonian-Laird method). Statistical heterogeneity was quantified with the I² statistic, with values >50% indicating substantial heterogeneity. Where applicable, subgroup analyses were planned a priori for pedicled flap subtype (PMMF vs other regional flaps such as SCAIF/SMIF), though insufficient stratified data limited pooled subgroup analyses.

For binary outcomes containing zero-event studies, a continuity correction of 0.5 was applied to studies with zero events in one treatment arm to allow calculation of risk ratios. Sensitivity analyses were performed using alternative zero-cell handling methods, including Mantel-Haenszel analyses without continuity correction, to assess the robustness of pooled estimates. All statistical analyses were conducted using standard meta-analysis software, with significance set at *p* < 0.05 (two-tailed).

## Results

### Study selection and characteristics

The search identified 391 records after duplicate removal. Following title and abstract screening, 86 full-text articles were assessed and 25 studies met all inclusion criteria (Fig. 1). The included studies, published between 1997 and 2025, encompassed 5114 patients undergoing reconstruction for a range of head and neck defects.

Eleven studies compared free flaps with the PMMF, historically the most common pedicled flap.^1^ Nine studies compared a type of submental flap and four studies evaluated free flaps versus the compared latissimus, deltopectoral and SCAIF. The remaining studies assessed a mixture of locoregional pedicled flaps (e.g. temporalis, trapezius) against various soft-tissue or osseous free flaps, most often the RFFF, ALT, free fibula or rectus abdominis. The flap subtypes are illustrated in Table 2. The indications for reconstruction were predominantly oncologic defects after head and neck cancer resection, although a few studies included patients with osteoradionecrosis or other benign conditions.

**Table 2:** Flap subtypes.

Source	Free Flap Subtypes	Pedicled Flap Subtypes
Al-Aroomi et al. (2025)^7^	SMAPF (n = 21)	SMGF (n = 16)
Benazzo et al. (2006)^8^	Jejunum (n = 47) Forearm (n = 5) ALT (n = 2)	PMMC (n = 9) Latissimus dorsi + pectoralis major (n = 1) Latissimus dorsi (n = 1)
Brown et al. (2009)^9^	RFFF (n = 28) Fibula (n = 5) Latissimus dorsi (n = 4) Scapula (n = 3) Rectus abdominus (n = 2) Osseous radial forearm (n = 2)	PMMC (n = 37) DP (n = 2) Latissimus dorsi (n = 2) Trapezius (n = 3)
Chen et al. (2005)^10^	RFFF (n = 28)	DP (n = 49)
Clark et al. (2009)^11^	Jejunal (n = 30) RFFF (n = 15) ALT (n = 11) Rectus abdominis (n = 5) Gastro-omental (n = 3)	PMMC (n = 67)
Escalante et al. (2021)^12^	RFFF (n = 68)	PMMC (n = 54) SCF (n = 18)
Forner et al. (2016)^13^	RFFF (n = 12)	SIF (n = 9) SCF (n = 2)
Gonzalez-Garcia et al. (2021)^14^	RFFF (n = 15)	SCAIF (n = 17)
Haidar et al. (2018)^15^	Myocutaneous (n = 62) Fasciocutaneous (n = 142)	Did not specify type
Hanasono et al. (2012)^16^	ALT (n = 70) Rectus abdominis myocutaneous (n = 11) Latissimus dorsi (n = 4) Serratus anterior (n = 1) Gracillis (n = 1) Groin (n = 1) Lateral arm (n = 1) RFFF (n = 1)	TMF (n = 27)
Howard et al. (2016)^17^	ALT (n = 9)	SIF (n = 16) Latissimus dorsi (n = 6)
Jang et al. (2013)^18^	ALT (n = 7) Rectus abdominis (n = 2) RFFF (n = 1) Latissimus (n = 1)	Pectoralis (n = 12) Trapezius (n = 1) SIF (n = 1)
Katna et al. (2021)^19^	ALT (n = 313) RAFF (n = 77) FOCF (n = 91)	PMMC (n = 134) PMMF (n = 13)
Katsnelson et al. (2021)^2^	Did not specify	Myocutaneous pedicled flap (n = 208)
Mahieu et al. (2016)^20^	Did not specify	Did not specify
McCrory, Magnuson (2009)^21^	Fibular (n = 4) Rectus abdominus (n = 8) RFFF (n = 22)	Platysmal (n = 6) PMMC (n = 18) Trapezius (n = 1)
Padha et al. (2022)^22^	Did not specify	PMMC
Patel (2020)^23^	RFFF (n = 89)	SIF (n = 57)
Paydarfar et al. (2011)^24^	RFFF (n = 33)	SIF (n = 27)
Pradhan et al. (2021)^25^ *Did not specify numbers	RFFF Fibular	PMMC DP
Ratnagiri et al. (2018)^26^ *Did not specify numbers for PF	Free fibula (n = 39) RFFF (n = 28) ALT (n = 19)	PMMC Latissimus dorsi DP
Sittitrai et al. (2023)^27^	RFFF (n = 59) ALT (n = 14)	PMMF (n = 23) SIF (n = 47) TMF (n = 16) SCF (n = 12)
Sittitrai et al. (2024)^28^	RFFF (n = 94)	SIF (n = 52)
Spoerl et al. (2019)^29^	RFFF (n = 230) ALT (n = 51) Free upper arm (n = 5) Latissimus dorsi (n = 25) Free fibula flap (n = 121) Deep circumflex iliac artery (n = 13) Scapular (n = 4)	PMMF (n = 40) SIF (n = 5)
Sun et al. (2025)^30^	Did not specify	Did not specify

Key:.

SMAPF: submental artery perforator flap, SMGF: submandibular gland flap, ALT: anterolateral thigh flap, PMMC: pectoralis major myocutaneous flap, RFFF: radial forearm free flap, DP: deltopectoral, SCF: supraclavicular flap, SIF: submental island flap, SCAIF: supraclavicular artery island flap, TMF: temporalis muscle flap, RAFF: radial artery forearm flap, FOCF: fibula osseo-cutaneous flap, PMMF: pectoralis major myofascial flap.

Patient demographics were broadly similar across groups, though several seven that pedicled flap cohorts tended to be older or have more comorbidities, suggesting selection bias toward using pedicled flaps in higher-risk patients.

### Outcomes: operative time

**Fig. 2:**
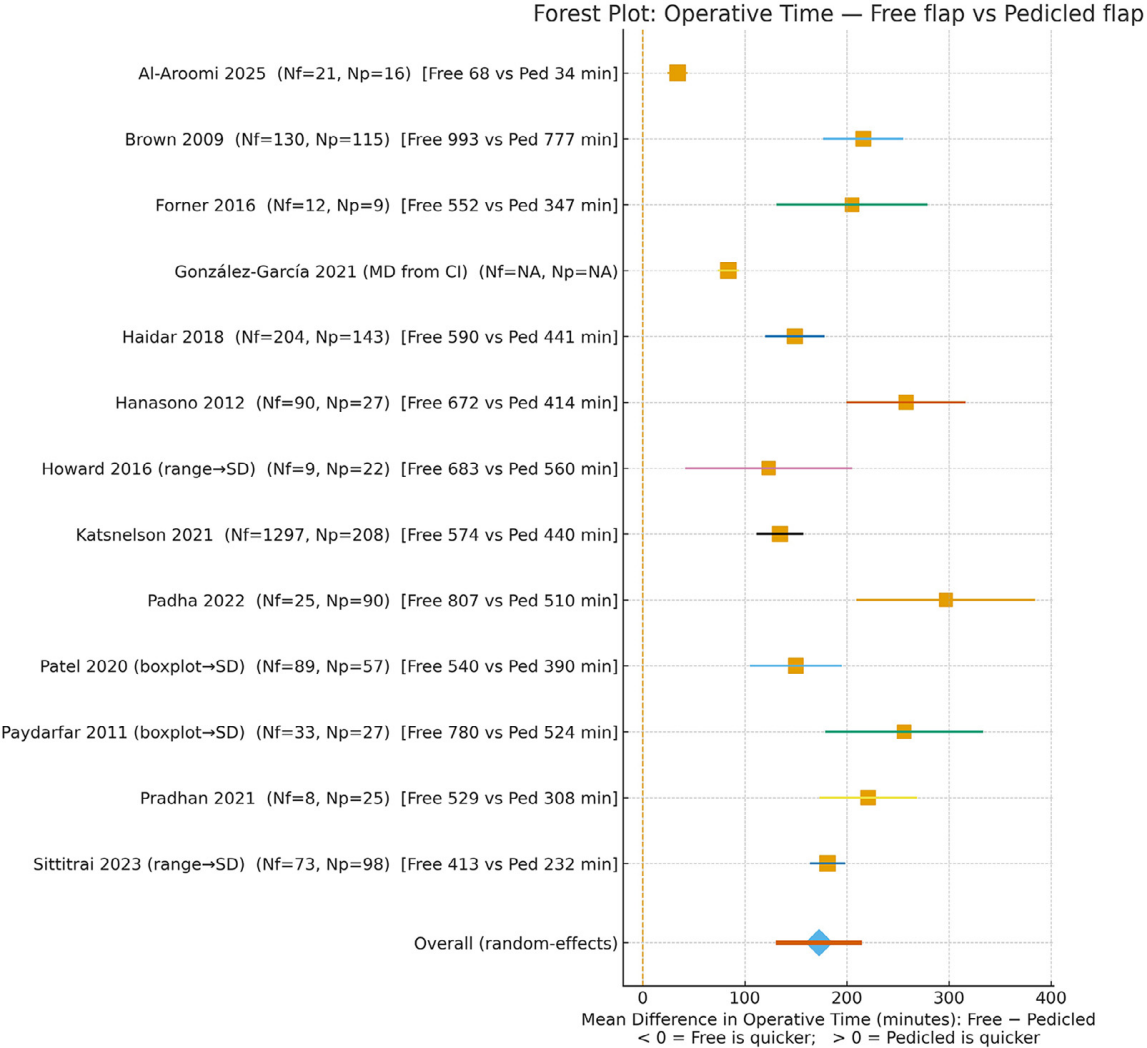
Comparison of operation time.

Considerable heterogeneity was observed for operative time across the twelve studies (I^2^ 98.8%, τ² 3950.91, χ² 519.36) (Fig. 2). The pooled mean difference demonstrated that free flap reconstruction required longer operative duration than pedicled flap reconstruction (+172.5 min, 95% CI 132.3–212.6). However, the 95% prediction interval was wide (+2.5 to +342.5 min), indicating substantial variability in effect size across studies. This suggests that while free flaps consistently required longer operative times, the magnitude of this difference varied considerably depending on flap type, defect complexity and institutional practice.

### Outcomes: hospital stay

**Fig. 3:**
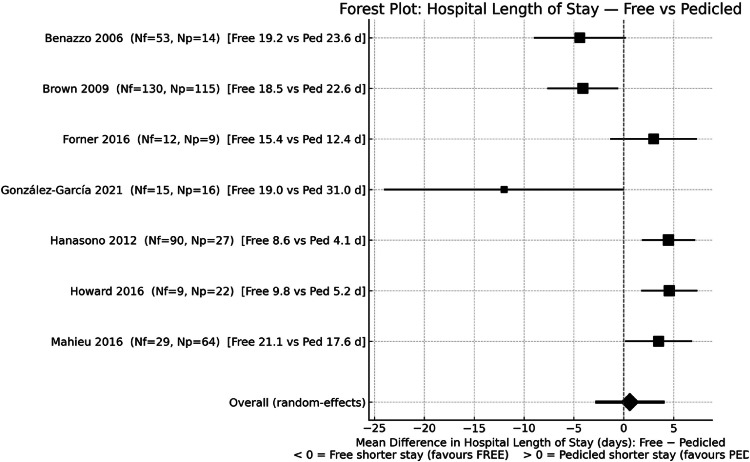
Comparison of LOS.

Seven studies reported postoperative LOS (Fig. 3). Results were mixed: three studies reported longer LOS with pedicled flaps and four reported longer LOS with free flaps. Pooled analysis showed no significant difference (MD +0.61 days, 95% CI −2.72 to +3.94; p = 0.72), indicating broadly comparable recovery durations. There was considerable heterogeneity in the data (I^2^ 85.90%, τ² 21.99, χ² 21.28).

### Outcomes: financial cost

**Fig. 4:**
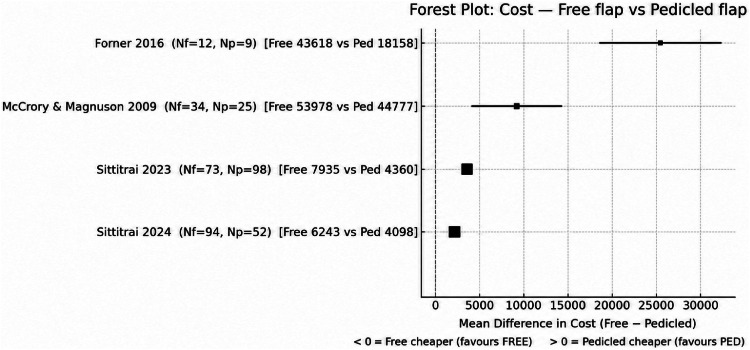
Comparison of financial cost.

All four studies evaluating cost found substantially higher expenditures associated with free flaps, between 1.3- and 3-fold higher than pedicled flap reconstruction (Fig. 4). The increased cost was attributed to longer operative time, microsurgical equipment and staffing and postoperative monitoring needs. Due to significant inter-system variability, a cost meta-analysis was not performed.

### Outcomes: surgical complications

1. *Flap Failure:* Fifteen studies reported on flap failure (Fig. 5). Complete flap loss was rare but occurred statistically more frequently in free flaps. The pooled RR was 1.65 (95% CI 1.30–2.09), indicating fewer flap failures with pedicled flaps. With continuity correction for zero-event studies, the results were similar (RR 1.60, 95% CI 1.25–2.04). There was moderate heterogeneity in the data (I^2^ 38.90%, τ² 0.198, χ² 22.91).

**Fig. 5:**
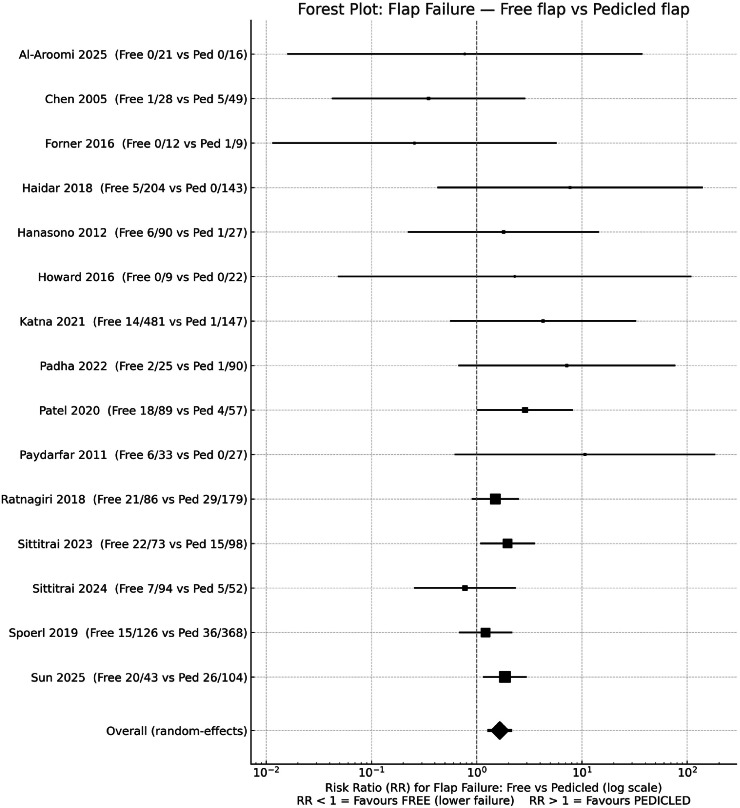
Comparison of flap failure rates.
2. *Wound Dehiscence*: Thirteen studies monitored wound dehiscence (RR 1.15, 95% CI 0.91-1.46), trending toward fewer events in pedicled flaps (Fig. 6). This difference did not reach statistical significance. The pooled meta-analysis with continuity correction was similar (RR 1.14, 95% CI 0.89-1.45). There was low heterogeneity in the data (I^2^ 18.8%, τ² 0.064, χ² 14.77).

**Fig. 6:**
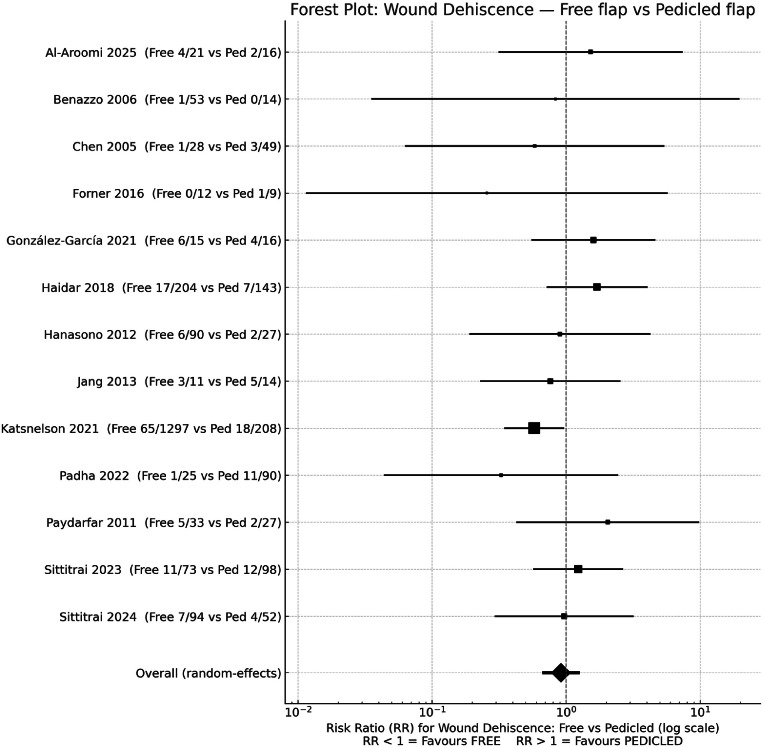
Comparison of wound dehiscence rates.
3. *Fistula Formation:* Twelve studies reported fistula formation (Fig. 7). The difference in results did not reach statistical significance (RR 0.83, 95% CI 0.43-1.62), trending toward fewer fistulas in free flaps. Following continuity correction for zero-event studies, pooled analysis was close to previous estimates (RR 0.83, 95% CI 0.43-1.59). There was low heterogeneity in the data (I^2^ 30.60%, τ² 0.142, χ² 15.84).

**Fig. 7:**
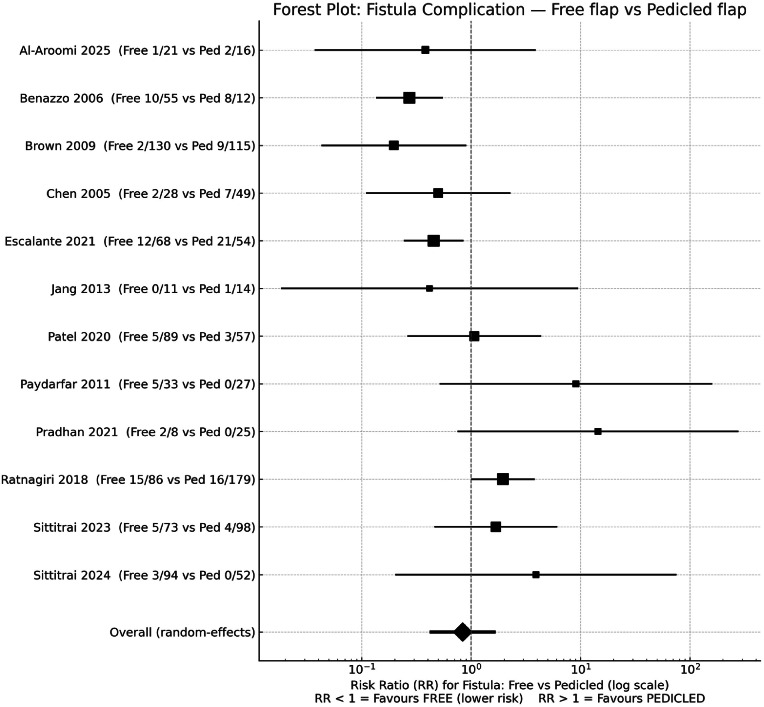
Comparison of fistula rates.
4. *Infection:* Twelve studies reported surgical site infection (Fig. 8). Pooled analysis demonstrated no statistically significant difference between free and pedicled flaps (RR 1.07, 95% CI: 0.78–1.48). Though, pedicled flaps showed a slight trend toward fewer infections. Accounting for continuity correction for zero-event studies, the results were similar (RR 1.05, 95% CI: 0.76–1.45). Heterogeneity was low (I^2^ 19.20%, τ² 0.058, χ² 13.62).

**Fig. 8:**
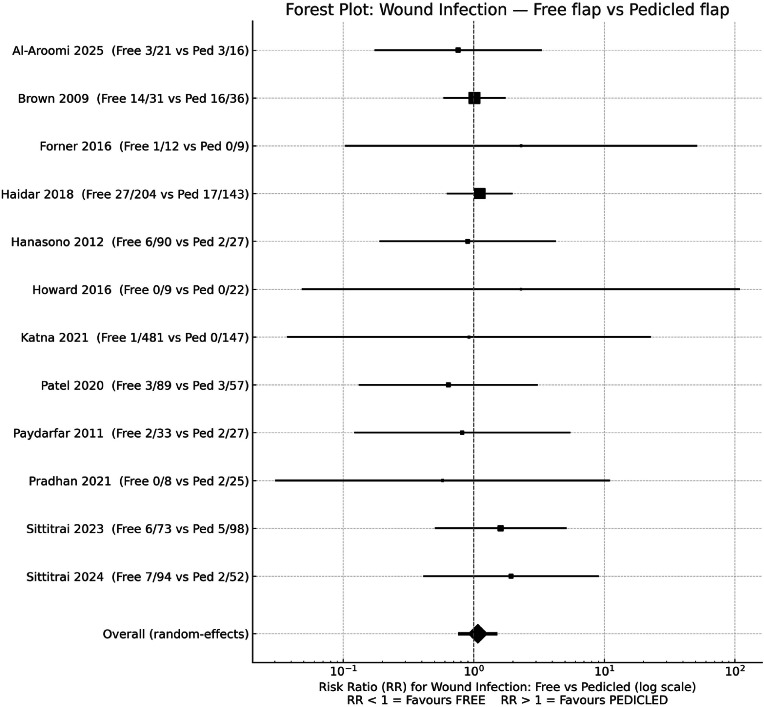
Comparison of wound infection rates.
5. *Wound Necrosis:* Eight studies compared necrosis rates (RR 0.76, 95% CI 0.32–1.82) (Fig. 9). This suggests a trend toward lower flap necrosis in free flap, though this difference was not statistically significant. The updated data allowing for continuity correction was similar (RR 0.79, 95% CI 0.34–1.84). There was substantial heterogeneity in the data (I^2^ 59.8%, τ² 0.441, χ² 17.43).

**Fig. 9:**
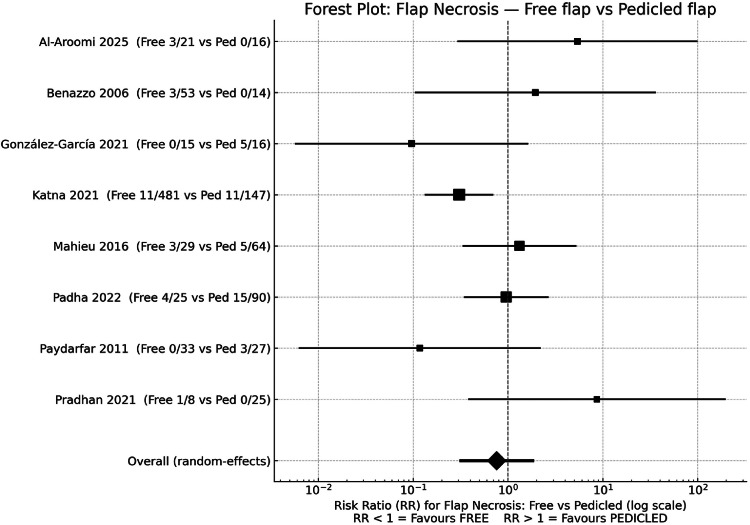
Comparison of wound necrosis rates.
6. *Need for Revision Surgery:* Twelve studies reported revision surgery (Fig. 10). Unplanned reoperation was more common in free flaps (RR 1.37, 95% CI 0.77–2.43). Following continuity correction for zero-event studies, pooled analysis showed a non-significant trend toward fewer revision surgeries in pedicled flaps (RR 1.41, 95% CI 0.79–2.51). Approximately 10–15% of free flap patients required vascular re-exploration, whereas pedicled flap revisions were infrequent and typically limited to minor wound management. There was considerable heterogeneity in the data (I^2^ 81.10%, τ² 0.892, χ² 58.14).

**Fig. 10:**
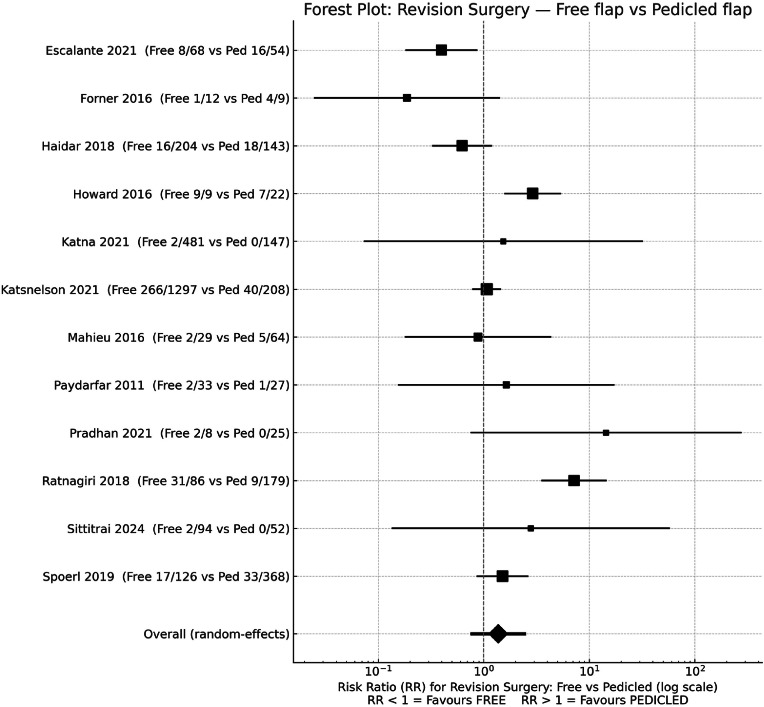
Comparison of revision surgery rates.

## Discussion

This systematic review and meta-analysis synthesised evidence from 25 comparative studies evaluating free versus pedicled flaps in head and neck reconstruction. The findings demonstrate the expected balance of advantages and limitations associated with each reconstructive approach.

Free flaps showed a trend toward reduced rates of fistula and tissue necrosis, reflecting the benefit of well-vascularised, pliable tissue tailored to complex defects. However, these advantages must be weighed against their clear drawbacks: substantially longer operative times, markedly higher costs and reliance on microsurgical expertise. Free flap reconstruction also carries the unique risk of microvascular failure, which accounted for the higher (though infrequent) need for re-exploration.

In contrast, pedicled flaps demonstrated several important practical advantages despite being technically less complex. They were associated with significantly shorter operative times, reduced resource utilisation and lower overall costs. Pedicled flaps also showed lower rates of several postoperative complications, including flap failure, wound dehiscence, infection and need for revision surgery, although most of these differences did not reach statistical significance. Notably, flap failure was statistically more common in free flap reconstruction, reflecting the inherent risk of microvascular compromise. Contemporary regional flaps, particularly the SCAIF and SMIF, achieved outcomes comparable to free flaps in selected small-to-medium defects. These modern techniques differ substantially from traditional pedicled options such as the PMMC, which consistently demonstrated higher rates of wound infection and dehiscence. Collectively, these findings suggest that newer pedicled flaps represent a reliable and clinically meaningful reconstructive alternative in appropriately selected patients.

Sensitivity analyses were performed using a continuity correction of 0.5 for studies with zero events in one or both arms. Recalculated pooled estimates for fistula, flap failure, wound dehiscence, infection, flap necrosis and revision surgery showed minimal changes compared with the original analyses, indicating that the results were robust to the method used for handling zero-event studies.

Patient selection remains central to flap choice. Elderly or medically frail patients may not tolerate the prolonged anaesthesia required for free flap reconstruction, making pedicled flaps a safer and more efficient option. In contrast, in medically fit patients with large or functionally critical oncologic defects, free flaps continue to provide superior long-term outcomes and reconstructive flexibility.

Cost considerations further support the value of pedicled options: across all cost analyses, free flaps were 1.3–3 times more expensive. Although high-volume microsurgical centres may offset some of these costs through improved outcomes and efficiencies, the economic burden remains substantial and reinforces the importance of nuanced, context-specific flap selection.

Finally, the absence of a significant difference in hospital stay suggests that both approaches, when successful, allow similar postoperative recovery trajectories.

### Limitations

Most included studies were retrospective and therefore subject to selection bias. Surgeons frequently selected pedicled flaps for older or comorbid patients, potentially inflating their complication rates. Conversely, free flaps were sometimes reserved for the most complex defects, introducing bias in the opposite direction. Variability in flap types, definitions of complications and perioperative protocols contributed to heterogeneity, limiting cross-study comparability. Although random-effects modelling was used, moderate-to-high heterogeneity persisted for several outcomes.

### Implications for practice

Free flaps remain the preferred option for large or complex defects in medically fit patients and in centres with established microsurgical capability. Pedicled flaps, however, demonstrated fewer complications overall (only flap failure was statistically significant), significantly shorter operative times and substantially lower costs. Modern regional flaps such as SCAIF and SMIF offer reconstructive outcomes approaching those of free flaps, strengthening their role in smaller defects, in medically frail patients and in resource-limited settings. The PMMC flap maintains value as a salvage or fallback option when microsurgery is not feasible.

### Future directions

The field of head and neck reconstruction continues to evolve. One clear need is for higher-level evidence. While an RCT might be difficult (ethical and practical issues in randomising to a possibly inferior reconstruction), prospective controlled studies or large registry analyses can help isolate the effect of flap choice. For instance, studies that carefully match patients by tumour stage and comorbidities and then compare outcomes of pedicled versus free flaps would be valuable. Also, emerging techniques such as free-style perforator flaps (which blur the line between pedicled and free, by using perforators and possibly anastomosing smaller vessels) might offer new solutions. Tissue engineering and three dimensional printing of grafts could one day challenge flaps, but for now, flaps remain superior.

Additionally, the cost issue invites future cost-effectiveness studies. With healthcare moving towards value-based care, demonstrating that the improved outcomes of free flaps justify their costs in terms of overall patient benefit is important. If certain pedicled flaps can achieve 90% of the outcome at 50% of the cost, there may be scenarios where that is an acceptable or even preferable trade-off. Thus, a nuanced approach, defect-driven and patient-driven flap selection, is the takeaway message, rather than a blanket statement that one technique is always superior.

## Conclusion

Free flaps remain the primary approach for extensive or complex head and neck defects; however, this review demonstrates that pedicled flaps are a highly effective alternative, offering shorter operative times, lower costs and fewer overall complications. Optimal reconstruction is not defined by a single superior technique but by matching the flap to the patient, the defect and the available institutional resources. When appropriately selected, both free and pedicled flaps can achieve excellent reconstructive and functional outcomes.

## Funding

None.

## Ethical approval

Not required.

## Declaration of competing interest

None declared.
